# The complete mitochondrial genome of the stink bug *Eocanthecona furcellata* (Hemiptera: Pentatomidae)

**DOI:** 10.1080/23802359.2021.1981169

**Published:** 2021-09-27

**Authors:** Yi Guo, Junjian Xiao, Dunsong Li, Juan Wang

**Affiliations:** a Plant Protection Research Institute, Guangdong Academy of Agricultural Sciences, Guangzhou, Guangdong, China; b College of Plant Protection, South China Agricultural University, Guangzhou, Guangdong, China; c College of Plant Protection, Shanxi Agricultural University, Taigu, Shanxi, China

**Keywords:** Mitochondrial genome, Heteroptera, Asopinae, Eocanthecona furcellata

## Abstract

The predatory stink bug *Eocanthecona furcellata* belongs to the subfamily Asopinae of Pentatomidae. In the current study, the complete mitochondrial genome of *E. furcellata* is determined. This mitogenome is 16,085 bp in size and comprises of 13 protein-coding genes, 22 transfer RNA genes, two ribosomal RNA genes, and a control region. Gene order is identical to that of the putative ancestral arrangement of insects. Nucleotide composition is biased toward A and T, which together made up 75.5% of the entire genome. All tRNAs have the clover-leaf structure except for the *tRNA^Ser(AGN)^* and the length of them ranges from 61 to 73 bp. The monophyly of Pentatomidae is highly supported by the phylogenetic tree and *E. furcellata* is very close to other carnivorous species of the remaining Pentatomidae species.

*Eocanthecona* Bergroth, 1915 belongs to the family Pentatomidae, a small genus with 20 known species in the world and 11 species in China (Zhao 2013). *Eocanthecona furcellata* (Wolff, 1801), as a common predator in south China, was massively reared in China as a biological agent. Widespread species occur from India and Sri Lanka through China and southeast Asia to Japan, the Philippines, and Indonesia (Rider and Zheng 2002). In this study, the complete mitochondrial genome of *E. furcellata* was sequenced and described. Adult specimens were collected from Heyuan city (24°6′30″N; 114°4′39″E) of Guangdong Province in China in 2021. Specimens were deposited in the Natural Enemy Insects Herbarium (accession number: GDPPRI-NI2021-22) of the Plant Protection Research Institute Guangdong Academy of Agricultural Sciences (GDPPRI) (Yi Guo, guoyi@gdaas.cn, Room 111, Plant Protection Building).

The total genomic DNA was extracted from the whole body of the specimen using the DNeasy Blood & Tissue Kit (Qiagen, Hilden, Germany) and stored at −20 °C until needed. The mitogenome was sequenced in BerryGenomics company that used NGS. One microgram of genomic DNA was used to generate libraries with an average insert size of 350 bp, which were sequenced using the Illumina HiSeq S6000 (San Diego, CA) with 150 bp paired-end reads on one sample per flow-cell lane. A total of 17,687,046 raw paired reads were generated. The quality of all sequences was checked using FastQC (http://www.bioinformatics.babraham.ac.uk/projects/fastqc). Clean reads were assembled and annotated using the MitoZ v2.4 pipeline (Meng et al. 2019).

The complete mitogenome of *E. furcellata* is 16,085 bp in size (GenBank accession number: MZ440302) including 37 typical insect mitochondrial genes (13 protein-coding genes, 22 transfer RNA genes, and two ribosomal RNA genes) and a control region. Gene order is identical to that of the putative ancestral arrangement of insects (Cameron 2014; Xu et al. 2019; Wang et al. 2021). The nucleotide composition of the mitogenome is biased toward A and T, with 75.5% of A + T content (A = 41.5%, T = 34.0%, C = 14.2%, and G = 10.3%). The AT-skew is positive (0.10) whereas GC-skew is negative (–0.16). Nine PCGs (*COII*, *COIII*, *ATP6*, *ND3*, *ND4*, *ND5*, *ND4L*, *ND6*, and *CYTB*) initiate with ATN codons, three PCGs (*COI*, *ATP8*, and *ND1*) initiate with TTG codons, and one PCG (*ND2*) initiates with TTA codon. The stop codons TAA and TAG are assigned to 11 and one protein-coding genes, respectively, whereas the TA residue is used by *ND5* as incomplete stop codon which is commonly in Heteroptera mitogenomes (Wang et al. 2017, 2021).

There are 22 tRNA genes, ranging from 61 to 73 bp in length, and all of them can be folded into typical clover-leaf secondary structure except for *tRNA^Ser(AGN)^*, the dihydrouridine (DHU) arm of which forms a loop, as is the case with most other insects (Jiang et al. 2016; Wu et al. 2020; Wang et al. 2021). The length of *IrRNA* and *srRNA* is 1340 bp and 790 bp, respectively. The A + T content of *IrRNA* and *srRNA* is 78.7% and 77.0%, respectively. The control region is located between *srRNA* and *tRNA^IIe^*, which is 1398 bp in length with an A + T content of 79.9%.

Maximum-likelihood (ML) tree was constructed based on sequences of 13 protein-coding genes and two rRNA genes from 13 species of different families and two outgroups by IQ-TREE 2.0.6 (Bui et al. 2020) under the GTR + I+G model (Figure 1). Each family showed a monophyletic cluster. The monophyly of the Pentatomidae was highly supported in this phylogenetic analysis, and predatory species were evolved from herbivorous species in the family Pentatomidae, which is also recovered in previous study (Wang et al. 2021). The complete mitogenome of *E. furcellata* could provide the molecular genetic markers for the further phylogenetic analysis in Pentatomidae.

**Figure 1.**
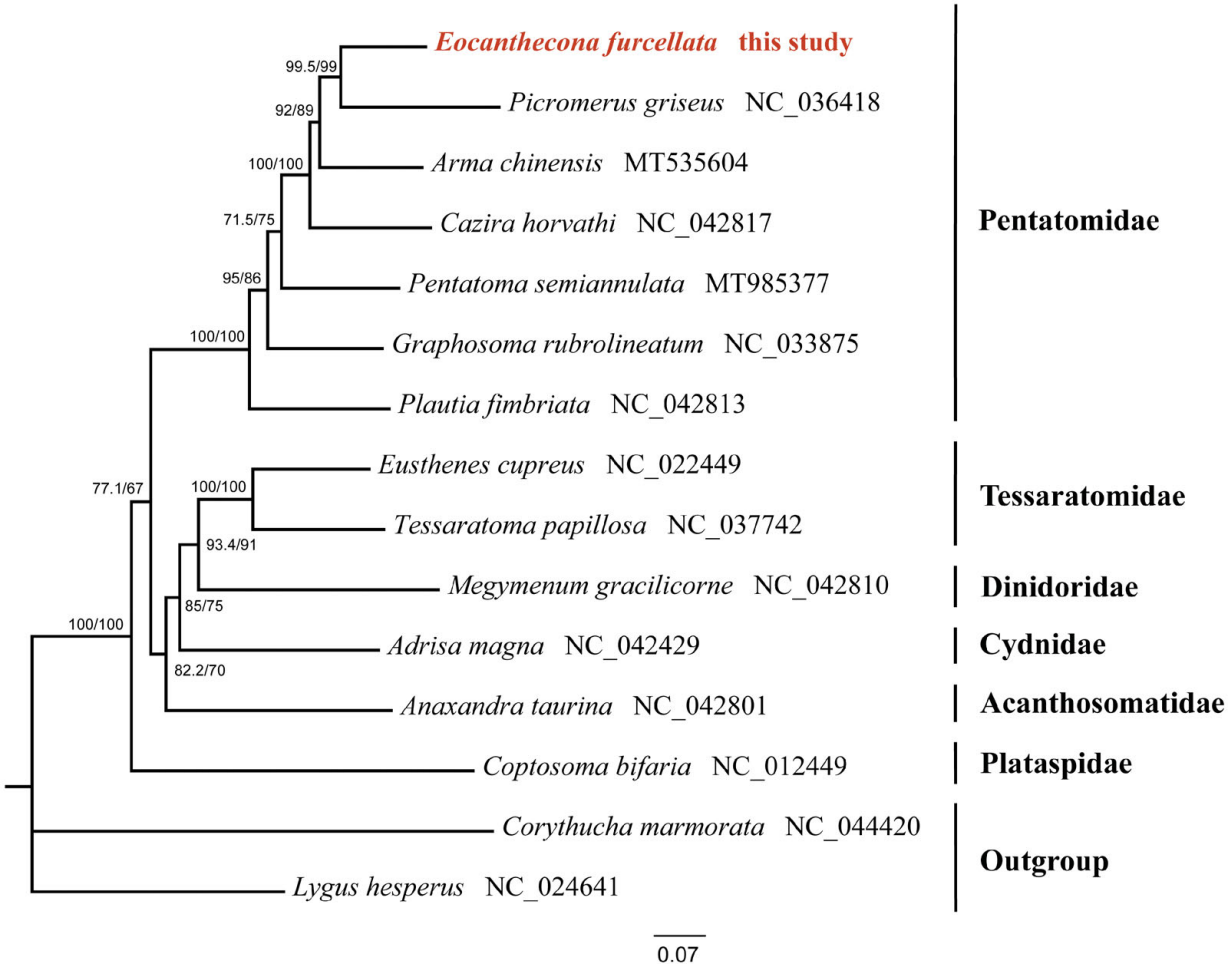
Maximum-likelihood (ML) phylogenetic tree of 13 Pentatomoidea species inferred from analysis of the 13 protein-coding genes and two rRNA genes. Numbers above each node separated by ‘/’ indicated support values of SH-aLRT (left) and ultrafast bootstrap (right). The newly sequenced mitochondrial genome was highlighted in red.

## Data Availability

The genome sequence data that support the findings of this study are openly available in GenBank of NCBI at https://www.ncbi.nlm.nih.gov/ under the accession no. MZ440302. The associated BioProject, SRA, and Bio-Sample numbers are PRJNA740352, SRR14901855, and SAMN19842624, respectively.

## References

[CIT0001] Bui Q, Schmidt H, Chernomor O, Schrempf D, Woodhams M, Haeseler A, Lanfear R. 2020. IQ-TREE 2: new models and efficient methods for phylogenetic inference in the genomic era. Mol Biol Evol. 37(5):1530–1534. 32011700 10.1093/molbev/msaa015 PMC7182206

[CIT0002] Cameron S. 2014. Insect mitochondrial genomics: implications for evolution and phylogeny. Annu Rev Entomol. 59(5):95–117. 24160435 10.1146/annurev-ento-011613-162007

[CIT0003] Jiang P, Li H, Song F, Cai Y, Wang J, Liu J, Cai W. 2016. Duplication and remolding of tRNA genes in the mitochondrial genome of *Reduvius tenebrosus* (Hemiptera: Reduviidae). Int J Mol Sci. 17(6):951. 10.3390/ijms17060951 PMC4926484 27322247

[CIT0004] Meng G, Li Y, Yang C, Liu S. 2019. MitoZ: a toolkit for animal mitochondrial genome assembly, annotation and visualization. Nucleic Acids Res. 47(11):e63. 30864657 10.1093/nar/gkz173 PMC6582343

[CIT0005] Rider D, Zheng L. 2002. Checklist and nomenclatural notes on the Chinese Pentatomidae (Heteroptera). I. Asopinae. Entomotaxonomia. 24(2):107–115.

[CIT0006] Wang J, Ji Y, Li H, Song F, Zhang L, Wang M. 2021. Characterization of the complete mitochondrial genome of *Pentatoma semiannulata* (Hemiptera: Pentatomidae). Mitochondrial DNA Part B. 6(3):750–752. 33763568 10.1080/23802359.2021.1875912 PMC7954408

[CIT0007] Wang J, Zhang L, Yang X, Zhou M, Yuan M. 2017. The first mitochondrial genome for the subfamily Podopinae (Hemiptera: Pentatomidae) and its phylogenetic implications. Mitochondrial DNA Part B. 2(1):219–220. 33473775 10.1080/23802359.2017.1310605 PMC7800415

[CIT0008] Wu Y, Yang H, Zhou W, Song F, Cai W, Li H. 2020. Characterization of the complete mitochondrial genome of *Arma custos* (Hemiptera: Pentatomidae). Mitochondrial DNA Part B. 5(3):2624–2626.

[CIT0009] Xu S, Wu Y, Cai W, Song F. 2019. The complete mitochondrial genome of the lychee stinkbug *Mattiphus splendidus* (Hemiptera: Tessaratomidae). Mitochondrial DNA Part B. 5(1):321–322. 33366538 10.1080/23802359.2019.1703609 PMC7748429

[CIT0010] Zhao Q. 2013. A revision of the Asopinae from China and the study of DNA taxonomy of *Arma*, *Carbula* and *Eysarcoris* (Hemipetra: Pentatomidae) [doctoral dissertation]. Tianjin, China: Nankai University.

